# Microbiological Ecological Surveillance of Zoonotic Pathogens from Hamadryas Baboons in Southwestern Saudi Arabia

**DOI:** 10.3390/microorganisms12122421

**Published:** 2024-11-25

**Authors:** Mohammed Abdullah Alqumber

**Affiliations:** Laboratory Medicine Department, Faculty of Applied Medical Sciences, Albaha University, Al Bahah 65779, Albaha, Saudi Arabia; maali@bu.edu.sa; Tel.: +966-55-220-9366

**Keywords:** *Papio hamadryas*, zoonotic infections, zoonosis, anthroponosis, pathogen coproprevalence

## Abstract

This study investigates parasitic and bacterial pathogens present in Hamadryas baboons (*Papio hamadryas*) and humans in southwestern Saudi Arabia. Fecal samples were collected from Hamadryas baboons (*n* = 999) from three city peripheries and humans from city centers (*n* = 1998) and peripheries (*n* = 1998) of southwestern and eastern Saudi cities. Parasitic examinations and bacterial cultures were conducted on these samples. Key findings include the identification of various parasitic and bacterial pathogens, with notable prevalences of *Staphylococcus aureus* (71.37% in baboons, 71.51% in humans), *Blastocystis hominis* (42.24% in baboons, 17.85% in humans), *Cryptosporidium* spp. (40.14% in baboons, 12.6% in humans), hookworms (37.44% in baboons, 18.57% in humans), *Strongyloides* spp. (37.34% in baboons, 17.39% in humans), *Enterobius vermicularis* (36.34% in baboons, 11.18% in humans), and *Campylobacter* spp. (29.73% in baboons, 1.86% in humans). Additionally, the prevalences of these microorganisms in human populations coexisting with baboons in southwestern city peripheries were 75.47%, 25.22%, 23.62%, 26.33%, 22.22%, 15.11%, and 3.8%, respectively. To further characterize bacterial isolates, *16S r*RNA gene sequencing was used, suggesting potential zoonotic and anthroponotic cycles. The results highlight significant pathogen prevalence among both baboons and human populations in proximity to baboon habitats, indicating a potential public health risk. However, shared environmental sources, such as contaminated water, were not thoroughly assessed and could play a role in pathogen transmission. The study’s focus on 18 different parasitic and bacterial pathogens allowed for the targeting of prevalent and indicative markers of zoonotic and anthroponotic transmission. In conclusion, these baseline data are crucial for the design of advanced studies to further investigate the zoonotic and anthroponotic transmission dynamics and the environmental factors influencing pathogen prevalence.

## 1. Introduction

As humans claim more wildlife land, and the contact between animals and humans becomes more prevalent, the risk of anthroponoses and zoonoses increases [1,2]. Zoonosis, a recognized global concern [3,4], underscores the capacity of pathogens to afflict multiple host species, giving rise to diverse human infections [5,6]. Indeed, the majority of human infectious diseases, ranging from 60% to 75%, originate from microorganisms that initially existed in non-human species [7]. Moreover, the percentage of helminthic infections afflicting humans that demonstrate zoonotic or zooanthroponotic potential can reach 95% [8,9]. Furthermore, the establishment of zooanthroponosis involving *Mycobacterium tuberculosis* and *M. bovis* highlights the bidirectional transmission between humans and animals [10]. This transmission of diseases between wildlife and humans is reported to be occurring at an increasing rate [11,12]. *Papio hamadryas* (hamadryas baboons) and other non-human primates can be infected and thus become carriers of human bacterial, viral, helminthic, and protozoan parasites [13,14,15,16,17,18,19], such as adenoviruses, flaviviruses, *Campylobacter* spp., *Salmonella* spp., *Giardia lamblia*, *Entamoeba histolytica*, *Hymenolepis* spp., *Enterobius vermicularis*, *Trichuris* spp., and hookworms [13,20,21]. 

Currently, zoonosis and anthroponosis may cause the emergence of new infectious agents or the persistence of known diseases in either population [1,2]. Southwestern Saudi Arabia is a known natural habitat for hamadryas baboons. These hamadryas baboon populations commonly overlap with the peripheries of human residential areas, while avoiding city centers [13]. The cities most severely affected by the presence of hamadryas baboons are Taif, Baha, and Abha, while other provinces of Saudi Arabia have no nearby hamadryas baboon populations [22]. The cities with overlapping habitats of hamadryas baboons and humans promote the occurrence of zoonotic and anthroponotic cycles, and thus pose a significant public health concern, potentially leading to an increased rate of infections [23]. The risk of infection associated with anthroponoses and zoonoses remains unclear in southwestern Saudi Arabia. An attempt to understand the ecology of infectious agents in the two populations can provide useful information on the public health risks associated with the presence of hamadryas baboon populations in Saudi Arabia. 

The coexistence of humans and wildlife presents challenges for global health. While human activities continue to encroach upon natural habitats, the risk of zoonotic and anthroponotic diseases is likely to escalate. Thus, proactive measures informed by scientific research and collaboration can help mitigate this risk and safeguard the health of both human and animal populations. By understanding the complex dynamics of disease transmission at the human–wildlife interface, we can work towards a more sustainable and resilient future for all species. With the aim of increasing the existing knowledge of this issue, the prevalence of 18 infectious agents in hamadryas baboons, as well as human populations living in hamadryas baboon-infested and hamadryas baboon-free cities was surveyed.

## 2. Materials and Methods

### 2.1. Sampling and Selection of Baboon Coprosamples

Freshly voided *Papio hamadryas* (PH) fecal specimens (*n* = 999) were procured from footpaths and parkland areas situated on the peripheries of three southwestern Saudi cities (Taif, Baha, and Abha) between July 2019 and December 2022, with 333 samples collected from each city. Collection was restricted to locales near mosques and areas that have a scheduled municipality cleaning to reduce cross-contamination. The collection of PH samples was 2 h after the daily cleaning to ensure fresh sample collection. Only newly extruded, intact, or contiguous fecal masses (fecal bolus), separated by ≥5 m from other fecal deposits and capable of yielding a minimum volume of 45 milliliters of final specimen, were included, to avoid cross-contamination of samples and support the individualistic representation of the samples.

### 2.2. Sampling and Selection of Human Coprosamples

**Figure 1:**
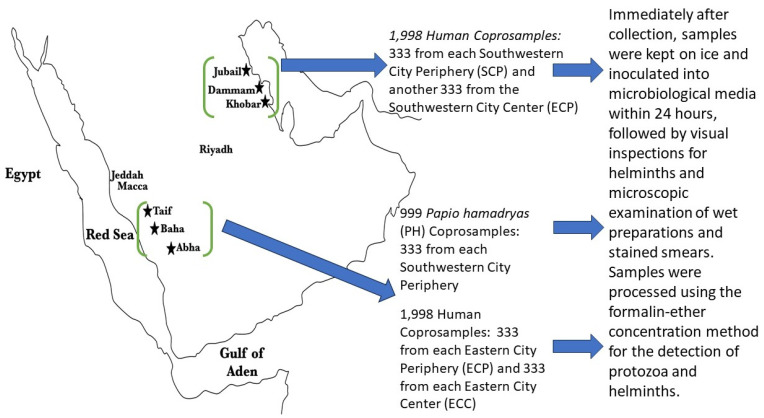
Map and flow chart for sample source and abbreviations indicating cities’ geographical locations where coprosamples were collected (not to scale). Legend: Samples collected included 1998 human coprosamples from the southwestern city periphery (SCP) and southwestern city center (SCC), with 333 samples from each location. Additionally, 999 *Papio hamadryas* (PH) coprosamples were collected from the southwestern city periphery, with 333 samples from each location. Another set of 1998 human coprosamples was collected from the eastern city periphery (ECP) and eastern city center (ECC), with 333 samples from each location.

Human coprosamples (*n* = 3996) were collected from the aforementioned southwestern cities (*n* = 1998), as well as from eastern cities (*n* = 1998) (Jubail, Dammam and Khobar), which are not infested with hamadryas baboons, thus serving as negative controls (Figure 1). In the southwestern region, human fecal samples were collected from toilet pans in mosques at city centers (SCC) and city peripheries (SCP) to investigate the potential transmission of pathogens. City centers are defined by high-density structures with at least 95% of land consisting of buildings, roads, and other urban infrastructure within a 1 km radius and fall within the Central Business District (CBD) boundaries. City peripheries are the areas surrounding the city boundary and are adjacent to natural, undeveloped land with minimal human activity. Human fecal samples were collected anonymously from male-only toilet facilities in mosques after the weekly Friday prayers, following morning cleaning, for logistical and cost reasons, as Friday prayers are obligatory for males but not females per Shari’ah Law. This procedure ensured that the collected samples were fresh and protected against cross-contamination. The collection process also minimized the possibility of sampling from the same individual multiple times. Although some repeated sampling from the same individual might occur if they attended mosque on different Fridays, this would still allow measurement of intraindividual variation, contributing to the broader understanding of the microbial burden in the community. Overall, the study did not distinguish between samples based on age, and it formed a part of a larger initiative by Albaha University to survey pathogenic microbes across the region. The samples represent the diversity of the male population, from children to adults. The fresh samples were collected between July 2019 and December 2022 for the location marked in Figure 1. This ecological, case–control, cross-sectional study design enabled the comparison of pathogen prevalence between areas with and without hamadryas baboons. Both human and baboon sample sizes for this study exceeded the numbers required to ensure optimal statistical power in detecting differences in pathogen prevalence. That is, a minimum of 97 samples per group were required to achieve 80% power at a 5% significance level, calculated using the sample size formula for comparing two proportions with *p*1 = 0.25 (presumed prevalence in one group (e.g., baboons)) and *p*2 = 0.1 (presumed prevalence in the other group (e.g., humans)):

(1) $$\frac{{\left(Z\frac{\alpha }{2}+Z\beta \right)}^{2}\times \left(p1\left(1-p1\right)+p2\left(1-p2\right)\right)}{{\left(p1-p2\right)}^{2}}$$

where Z_α/2_ and Z_β_ are the critical values for the chosen significance level and power, that is, 1.96 and 0.84, respectively. Alpha (α), the probability of rejecting the true null hypothesis (type I error), is the statistical significance threshold, set at 0.05, indicating a 5% risk of a false positive. Beta (β) is the probability of failing to reject the false null hypothesis (type II error) and is related to test power, which is the probability of correctly rejecting a false null hypothesis (1 − β). It is set at 80%, corresponding to a beta of 0.20, indicating a 20% chance of missing a true effect [24,25].

### 2.3. Microbial Analysis of Coprosamples

Immediately after collection, the samples were kept on ice, and within the next 24 h, microbiological media were inoculated, as described below. Afterwards, naked-eye visual inspection of the samples was performed to determine the presence of adult helminths. Next, wet preparations of fecal samples, and smears stained with eosin, iodine, and Ziehl–Neelsen were examined microscopically. The samples were subsequently processed by the formalin–ether concentration method using a Fecal Parasite Concentrator kit (Evergreen Scientific, Los Angeles, CA, USA). More specifically, 4 g of stool was mixed with 7 mL 10% formol water, and was centrifuged at 1000× *g* with 3 mL diethyl ether, after which it was vortexed for 1 min and centrifuged again for 10 min. Next, the fecal debris, ether, and formol water were decanted and each sample observed under a microscope at 100× and 400× magnification (Olympus BX46 Clinical Microscope, Evident Corporation, Tokyo, Japan). The study examined 18 different pathogens to assess zoonotic risks related to hamadryas baboon populations in southwestern Saudi Arabia. These pathogens encompassed bacteria (*Campylobacter* spp., *Clostridioides difficile*, *Mycobacterium* spp., *Salmonella* spp., *Shigella* spp., *Staphylococcus aureus*), protozoa (*E. histolytica*, *G. lamblia*, *Cryptosporidium* spp., *Cyclospora* spp., *Balantidium coli*, *Blastocystis hominis*), and helminths (*E. vermicularis*, *Hymenolepis* spp., and hookworms, *T. trichiura, Schistosoma mansoni, Strongyloides stercoralis*). The selection criteria were based on their zoonotic potential and their impact on various body systems with differing degrees of clinical severity. Furthermore, some of these microorganisms, including *S. aureus*, *C. difficile*, *Bl. hominis*, and *E. histolytica*, were selected because they are known to exist as part of the normal human intestinal flora (commensals), thereby ensuring their prevalence is not influenced by individuals seeking medical treatment. Representative of isolated pathogens, that is, a randomly selected subset of the identified pathogens, underwent laboratory testing and genetic sequencing to determine their prevalence and genetic diversity in both human and baboon populations. Samples were inoculated on mannitol salt agar, MacConkey agar, Skirrow’s or Campy-BAP agar, and Lowenstein–Jensen media (Becton Dickinson and Company, Riyadh, Saudi Arabia) for *S. aureus*, Enterobacteriaceae, *Campylobacter* spp., and *Mycobacterium* spp., respectively. For clostridia, coprosamples weighing 10 g each underwent a 10 min heating process in a water bath set at 70 °C. Subsequently, loopful inoculum was plated on meat infusion agar, incorporating 10% lactose, neutral red, 10% of a mixture of egg yolk and normal saline, and 0.1% sodium thioglycolate, as described by Willis and Hobbs (1958) [26], and incubated at 37 °C anaerobically (90% H_2_ and 10% CO_2_) for 24 h in anaerobic jars. Next, colonies were examined macroscopically and microscopically and their morphologies recoded. An L-proline aminopeptidase activity test (Prodisk; Remel, Lenexa, KS, USA) was used for clostridia. Columbia sheep blood agar (CSB) was used as a positive control to ensure that the culture conditions allowed for bacterial growth and the collected stool samples contained viable bacteria. Presumptive pathogens were confirmed using basic microbiological tests, API 20E strips (BioMerieux, Salt Lake City, UT, USA), or *16S r*RNA gene sequencing, as described below. 

### 2.4. Molecular Characterization of Albaha City Isolates Using 16S rRNA Gene Sequencing

Several Albaha city isolates from each type of selective media, assumed to correspond to presumptively different strains as judged by colony morphology, of *Campylobacter* spp., *Clostridioides* spp., *Mycobacterium* spp., and *S. aureus* were subjected to *16S r*RNA gene amplification, and the resulting PCR products were run on a gel, purified via a QIAquick Gel Extraction Kit (QIAGEN, Hilden, Germany), sequenced, and BLASTN analyzed (https://www.ncbi.nlm.nih.gov/, accessed on 1 January 2024). DNA extraction was performed using a Qiagen DNeasy tissue kit (QIAGEN, Hilden, Germany) according to the manufacturer’s instructions, using 4 h logarithmic growth phase cells in Todd–Hewitt Broth (Difco, Beyrouth, Lebanon). The *16S* primers and the PCR amplification conditions were identical to those described previously [27]. Sequencing was performed using the ABI Prism kit and the ABI 3100 DNA sequencer (BigDye terminator sequencing kit, AmpliTaq DNA polymerase FS, GeneAmp PCR system 9700; ABI, Tampa, FL, USA), according to the manufacturer’s instructions. Finally, the similarities of the obtained sequences to those of known species were determined using the BLASTN program. Isolated strains with ≥99% *16S r*RNA gene homology were grouped together as a cluster designated with the name of the nearest *16S r*RNA gene BLASTN match obtained from the website (https://www.ncbi.nlm.nih.gov/, accessed on 1 January 2024). 

### 2.5. Randomized Sampling of Primary Health Centers for Clinical Correlations

All Primary Health Centers within each surveyed locale, designated with a distinct identifier, were randomized using Google’s random number generator (https://www.calculator.net/random-number-generator.html, accessed on 1 January 2024). A subset of 20 centers was selected to ensure impartiality. Physicians from these centers were contacted via telephone to collect the data on the distribution of respiratory infections compared to gastrointestinal infection. One standardized question was utilized to ascertain this distribution, which was “What is the ratio of respiratory to gastrointestinal infections among the cases you have seen in your Primary Health Center?”.

### 2.6. Statistical Analysis of Pathogen Prevalence

To assess the significance of differences in pathogen prevalence among the samples collected from various locations, a one-tailed *Z*-test for two population proportions was conducted. This statistical approach was chosen due to the directional hypothesis that samples from locations infested with hamadryas baboons would exhibit a higher prevalence of zoonotic pathogens compared to those from baboon-free areas. The one-tailed test allows us to determine whether the prevalence in one group is significantly greater than in another. The *Z*-test was performed using SPSS (version 14; 2005 SPSS Inc., Chicago, IL, USA) software, with a significance level set at *p* < 0.05. 

## 3. Results

### 3.1. Baboon Demographics and Pathogen Prevalence Rates

The baboons surveyed exhibited similar pathogen prevalence rates across their diverse habitats, from rocky cliffs to peripheral suburbs, as found in previous studies [13], identifying them as potential reservoirs for infectious agents. Direct observation estimated rural suburb baboon population densities as 7, 8, and 10 individuals/km² for Taif, Baha, and Abha, respectively. From a total of 999 baboon and 3996 human fecal samples, six protozoan taxa, six helminth taxa, and six bacteria taxa were isolated and identified, as detailed in Table 1. Furthermore, two bacteria (*C. difficile* and *S. aureus*), four protozoa (*E. histolytica*, *G. lamblia*, *B. coli*, *Bl. hominis*), and four helminths (*E. vermicularis*, *T. trichiura, Sch. mansoni*, *St. stercoralis*) were identified at the species level. *S. aureus* dominated both PH and human samples, with rates exceeding 68.7% across all regions. *Bl. hominis*, *Cryptosporidium* spp., hookworms, *St. stercoralis*, *E. vermicularis*, and *Hymenolepis* spp. were among the most prevalent isolates. 

### 3.2. Comparative Analysis of Infectious Agent Prevalence in Baboon and Human Fecal Samples

The analyses revealed that the average rate of prevalence of infectious agents was significantly higher in the PH fecal samples compared to that found in human fecal samples (*p* = 0), except for *S. aureus*, which was only slightly more prevalent in PH samples (71.37%) than human samples (71.17%), but this difference was not significant (*p* = 0.41). In southwestern cities, the SCP samples exhibited statistically significantly higher prevalence rates for most pathogens compared to human SCC samples, except for *Sch. mansoni* (*p* = 0.9) and *Salmonella* spp. (*p* = 0.13). Conversely, in eastern cities, there were no significant differences in the occurrence rates between fecal samples from city centers (ECC) and peripheries (ECP), except for *Hymenolepis* spp. (*p* = 0.01) (Table 1 and Table 2).

### 3.3. Molecular Diversity of Pathogens Among Baboon and Human Samples 

*C. difficile* isolates subjected to *16S r*RNA gene sequencing (*n* = 22) included eight cultivated from PH, eight from SCC, and six from SCP samples. Five of these—four cultivated from PH and one from SCC samples—had a unique *16S r*RNA gene sequence and did not cluster with any other isolates. Two clusters corresponding to *C. difficile* strain CD196 and *Clostridium* sp. CYP4 comprised isolates cultivated from the three sample types (Table 3). The three remaining clusters—*C. difficile* strain JCM 5256, *C. difficile* strain JCM 5244, and *C. difficile* strain 630—comprised isolates cultivated from two sample types, i.e., either PH and SCP, or SCC and SCP samples (Table 3). Representative *S. aureus* isolates subjected to *16S r*RNA gene sequencing (*n* = 46) coalesced into nine clusters. Four *16S r*RNA gene clusters were shown to be unique to one specific sample type, which did not cluster with any other isolates cultivated from any other sample type (Table 3). Two clusters, designated *S. aureus* strain 502A and *S. aureus* strain P91-7354b, comprised isolates obtained from each of the three sample types. Two further clusters, *S. aureus* strain USA-ISMMS1 and *S. aureus* strain SMKV-2, comprised isolates cultivated from either PH and SCP, or SCC and SCP samples (Table 3). One *Campylobacter* spp. *16S r*RNA gene cluster, *Ca. jejuni* subsp. *jejuni* 81-176, comprised isolates cultivated from the three sample types. Three further *Campylobacter* spp. clusters comprised isolates cultivated from PH and SCP samples. In addition, the cluster designated *Ca. fetus* subsp. *venerealis* strain 84-112 comprised isolates cultivated from PH and SCC samples. The remaining six clusters comprised isolates cultivated from only one sample type (Table 3). A total of 40 *Mycobacterium* spp. isolates coalesced into nine clusters (Table 3). Three of these clusters comprised isolates cultivated from all three sample types, while two clusters comprised isolates cultivated from PH and SCP samples. The remaining clusters comprised isolates cultivate from only one sample type (Table 3). 

### 3.4. Regional Differences in Pathogen Prevalence

Samples were collected between July 2019 and December 2022, spanning multiple seasons over three years. Infections caused by *Campylobacter* spp., *Salmonella* spp., and *Shigella* spp. were 18.32%, 15.59%, and 14.93% more prevalent, respectively, during the warmer months (June–December) with *p*-values of 0.00158, 0.00152, and 0.00203. Conversely, *E. histolytica, G. lamblia, Cryptosporidium* spp., *Cyclospora* spp., *B. coli, Bl. hominis*, and hookworm infections were more prevalent during rainy seasons (November to May) with *p*-values of 0.0047, 0.0032, 0.0069, 0.0083, 0.0021, 0.0098, and 0.0076, respectively. No such significant seasonal variation was observed for *S. aureus* (*p* = 0.485), *C. difficile* (*p* = 0.742)*, Mycobacterium* spp. (*p* = 0.621), *E. vermicularis* (*p* = 0.913)*,* or *Hymenolepis* spp. (*p* = 0.567).

### 3.5. Clinical Correlations

A survey of 240 Primary Health Centers was conducted, with 40 centers sampled from each of the study’s six cities, comprising 20 centers from the city centers and 20 from the peripheries. The aim was to ascertain the ratios of respiratory to gastrointestinal infections. The hypothesis posited that regions where human and hamadryas baboon habitats overlap might experience elevated gastrointestinal infections due to potential zoonotic transmission. Conversely, areas with higher levels of human-to-human contact and no baboon populations were expected to exhibit increased respiratory infections. The study revealed average respiratory to gastrointestinal infection ratios of 7.81:1, 7.19:1, 6.45:1, and 5.25:1 for ECS, ECP, SCC, and SCP, respectively.

## 4. Discussion

Zoonoses and anthroponoses between animals, including primates, and humans could cause the emergence of pathogens [4,5,6,7]. Helminths found to infect humans comprise many that are zoonotic [8,9], and *M. tuberculosis* anthroponosis in South Africa has already been proven [28]. Hamadryas baboons, and other non-human primates, can be infected and thus become carriers of human pathogens [13,29], such as *Escherichia coli*, *E. histolytica*, *Hymenolepis* spp., *G. intestinalis*, *E. vermicularis*, *Ascaris* spp., *Trichuris* spp., and hookworms [13,30,31,32]. Therefore, it could be hypothesized that a cycle of zoonoses and anthroponoses may result in a higher rate of infections in areas where the habitats of the two populations overlap, such as city peripheries in southwestern Saudi Arabia. 

A coprological examination of fecal samples from the involved populations was conducted to measure the prevalence of 18 different pathogens with the aim of establishing whether living at the peripheries of southwestern cities in Saudi Arabia, where hamadryas baboons dwell, is statistically associated with a significantly higher infection rate with the surveyed hamadryas baboon-carried pathogens compared to residing in hamadryas baboon-free locations. The investigation used eastern cities as a negative control, since they are free of hamadryas baboons, to mitigate any potential confounding factors associated with city peripheries other than hamadryas baboons in the tested southwestern cities. The obtained results revealed significantly higher pathogen prevalence in the hamadryas baboon fecal samples compared to the human samples for all tested pathogens except the normal human floral pathogen *S. aureus*, which was found to exist in humans, regardless of the presence of hamadryas baboon populations [33,34,35]. *16S r*RNA gene analysis revealed that half of the sixteen human SCP *S. aureus* isolates analyzed fitted within clusters containing *Papio hamadryas* and human SCC *S. aureus* isolates. In addition, half of the remaining human SCP isolates were incorporated with human SCC isolates in two clusters, and all SCC *S. aureus* isolates also clustered with isolates from other sample types (Table 3). These findings indicate that the *S. aureus* community is shared and swapped between the three studied populations. Similar clustering patterns were observed for *Campylobacter* spp., *Mycobacterium* spp., and *Clostridium* spp. isolates, which were also found across all three populations (Table 3). Furthermore, the prevalence of all tested pathogens (*n* = 18), except for *Sch. mansoni*, was notably higher in human fecal samples collected from SCP than those from SCC. These findings support to the hypothesis that hamadryas baboons may have an influence on infection rates among the SCP human population, supporting previous studies suggesting that zoonotic and anthroponotic transmission is possible in areas where human–nonhuman primates overlap [32,36,37]. In addition, the Central Department of Statistics and Information of Saudi Arabia (http://www.cdsi.gov.sa, accessed on 1 January 2024)) show no significant differences between the living standards of ECP and SCP populations. This indicates that reasons other than hamadryas baboons are unlikely to be associated with the higher prevalence rate of pathogens in human SCP samples. In eastern cities, unlike hamadryas baboon-infested southwestern cities, the ECP samples did not exhibit significantly higher prevalence rates for any of the tested pathogens, with the exception of *Hymenolepis* spp., showing 12.31% and 16.22% prevalence rates for ECC and ECP, respectively. This discrepancy may be attributed to various reservoirs and carriers existing in ECP environments for *Hymenolepis* spp., such as arthropods, mainly beetles, as intermediate hosts, and small mammals, such as rodents, as definitive hosts; hence, the prevalence rate of *Hymenolepis* spp. in SCP (24.92%) is significantly higher (*p* < 0.0001) than in ECP (16.22%) [38].

Serovars of *Salmonella*—such as *S. enterica* subsp. *enterica* serovar Typhi and serovar Paratyphi—are highly adapted to humans and have no other known natural hosts, while others, such as *Salmonella* serovar Typhimurium, have a broad host range and infect a wide variety of animal hosts [39]. Therefore, the observed lack of correlation (Table 2) between the carriage rate of this pathogen in hamadryas baboons and its prevalence in human samples may be due to the host specificity of the different *Salmonella* serovars present in the tested populations [40,41]. 

*Sch. mansoni* transmission requires the existence of suitable snails releasing cercariae, which have a short life. Moreover, a water body from which the cercariae can penetrate the skin of potential hosts is a prerequisite for an infection to occur [42,43]. Consequently, the mere contamination of hands or food with fecal-polluted soil material is inadequate for *Sch. mansoni* transmission. Therefore, simply residing in areas populated by hamadryas baboons would not result in an increased rate of infection with this trematode pathogen. This is important to acknowledge, given that hamadryas baboons are already considered a maintenance host for this pathogen in Saudi Arabia [13,44]. As previously noted, a shared water body between the two populations needs to exist—a condition that was not met in any of the sampled areas.

Clinical observations collected by focus interviews align with the results of this research, indicating that eastern cities exhibit a higher prevalence of human-to-human infectious diseases (respiratory infections) transmitted through direct or indirect contact or droplets, while western cities generally report a higher prevalence of gastrointestinal infections. For example, an analysis of demographic data of Middle East respiratory syndrome-related coronavirus (MERS-CoV) cases reveals that the Eastern Province of Saudi Arabia was more affected than the Southern Province, which was the least affected region in the country [45]. Moreover, a community-based study in the Baha region found that 21.1% of 19,939 children harbored intestinal parasites [46]. In contrast, a study in the Eastern Province found that 10.44% of 3258 primary school children aged 6–11 years were asymptomatic carriers of *Salmonella* spp., *Shigella* spp., and intestinal parasites, with a 9.30% prevalence of parasitic infection [47]. This higher prevalence of human-to-human infectious diseases in eastern cities can be attributed to their dense populations being major economic and industrial hubs, attracting a diverse population with various backgrounds, creating favorable conditions for the transmission of infectious agents among humans. Studies have identified that urbanization and increased building density can increase the risk of spreading infectious diseases as a significant driver of infectious disease prevalence, particularly respiratory diseases, due to increased human-to-human contact [48]. This demographic diversity contributes to a broader spectrum of infectious diseases, as different pathogens may be introduced by individuals traveling to the region for work or business [3,4]. The western cities’ unique ecological setting creates a high risk of infectious agent exposure for humans and baboons due to their close proximity. Factors such as the region’s mountainous terrain and diverse ecosystems, which house diverse wildlife populations like baboons, contribute to this risk. This close human–wildlife interaction in southwestern Saudi Arabia underscores the importance of disease surveillance and prevention. The analysis of 18 zoonotic pathogens reveals various disease risks tied to urbanization, the contamination of farms and food sources by baboons, and increased human–baboon interactions due to inadequate waste management practices, as per communications with local authorities. However, our study’s limitations, like reliance on fecal samples and the study’s design, highlight the need for further longitudinal and experimental research to fully understand the associated health risks. While the *Z*-test sheds light on pathogen prevalence variations, future surveys should consider factors like age, sex, and socioeconomic status for a more thorough analysis. To address these issues, urban planning strategies should minimize human intrusion into natural habitats and include green corridors and buffer zones to create separation from baboons, aiming to reduce direct contact. Sustainable agricultural practices, awareness campaigns, and eco-friendly waste management methods are crucial for eliminating attractions for baboons. Baboon-resistant crop protection measures can also help mitigate crop foraging [Personal communication with Prof. Abdullah Saleh Al-Ghamdi, Chair of the Sheikh Said Ben Ali Alangari for Olive Research at Albaha University, Albaha, Saudi Arabia]. 

## 5. Conclusions

Our study emphasizes the importance of regular surveys and disease monitoring to promptly identify health risks and guide interventions in areas where human–baboon interactions occur. While our findings shed light on the prevalence of pathogens carried by hamadryas baboons and their potential impact on human health, the study’s limitations, such as reliance on fecal samples and the cross-sectional design, emphasize the necessity for further longitudinal research and experimental studies to comprehensively understand the associated health risks. It is crucial to consider the potential role of shared environmental factors, such as contaminated water sources, which may contribute to pathogen transmission. Moreover, future surveys would benefit from additional analysis that considers confounding variables like age, gender, and socioeconomic status to enhance robustness. Recommended solutions encompass urban planning strategies aimed at minimizing the intrusion of human settlements into natural habitats, implementing sustainable agricultural practices, and adopting eco-friendly waste management methods to mitigate risks associated with human–wildlife interactions. By addressing these challenges and leveraging insights from our study, we can advance disease surveillance and prevention efforts in regions where human and wildlife populations intersect, ultimately safeguarding public health and promoting coexistence between humans and baboons.

## Figures and Tables

**Table 1:** Prevalence rates (%) for each surveyed pathogen among different studied populations.

	Baboon Samples			SCP Samples			SCC Samples			ECP Samples			ECC Samples		
Infective Agent	Taif	Baha	Abha	Taif	Baha	Abha	Taif	Baha	Abha	Jubail	Dammam	Khobar	Jubail	Dammam	Khobar
*Campylobacter* spp.	29.73	29.43	30.03	3.90	3.90	3.60	1.50	1.80	1.50	0.90	0.60	0.90	0.60	0.90	0.30
*Clostridioides difficile*	29.13	28.53	28.83	6.01	5.71	6.31	2.70	3.00	3.00	1.80	2.10	1.80	1.50	2.10	1.80
*Mycobacterium* spp.	24.62	24.32	24.62	2.70	2.40	3.00	1.20	0.90	1.50	0.60	0.60	0.60	1.20	0.90	1.20
*Salmonella* spp.	11.11	11.41	10.81	3.00	2.70	3.30	2.10	2.40	2.10	1.80	2.10	1.80	2.40	2.40	1.80
*Shigella* spp.	11.41	11.11	11.11	9.61	9.31	9.61	4.50	4.80	4.20	2.70	2.40	2.40	3.00	2.40	2.70
*Staphylococcus aureus*	71.77	70.87	71.47	75.68	74.77	75.98	70.27	69.37	70.27	69.37	69.97	68.77	69.07	71.77	68.77
*Entamoeba histolytica*	15.92	16.22	15.92	6.91	6.61	7.21	3.30	3.00	3.60	1.80	1.80	2.10	2.40	2.10	1.50
*Giardia lamblia*	24.32	24.32	24.62	3.90	3.60	3.90	2.40	2.10	2.40	0.00	0.90	0.60	0.00	0.60	0.30
*Cryptosporidium* spp.	39.64	39.94	40.84	23.72	23.42	23.72	9.91	9.31	9.61	7.21	6.61	6.31	6.91	7.21	6.31
*Cyclospora* spp.	34.83	34.23	34.53	15.92	15.62	16.22	8.11	8.71	8.41	6.31	6.91	6.91	6.01	6.31	6.31
*Balantidium coli*	23.72	23.12	24.02	12.31	12.91	12.91	9.31	9.01	9.31	6.91	7.51	6.61	7.21	7.51	7.51
*Blastocystis hominis*	42.64	41.74	42.34	24.92	25.53	25.23	17.12	17.42	16.82	14.41	13.51	13.51	13.21	12.61	12.91
*Enterobius vermicularis*	35.74	36.34	36.94	14.71	15.62	15.02	11.11	10.51	11.41	9.01	8.71	8.71	8.71	8.71	8.41
*Hymenolepis* spp.	35.44	36.04	36.34	25.23	24.62	24.92	16.22	15.62	17.72	16.52	16.22	15.92	12.31	12.61	12.01
Hookworms	37.54	37.24	37.54	25.83	26.43	26.73	16.82	15.92	17.12	14.41	14.41	14.71	14.11	14.71	14.41
*Trichuris trichiura*	28.83	28.83	29.13	24.02	24.92	24.62	14.11	14.71	14.41	11.71	12.31	11.71	11.41	12.01	11.41
*Schistosoma mansoni*	20.72	20.72	21.02	0.30	0.60	0.60	0.90	1.20	0.90	0.00	0.60	0.30	0.60	0.60	0.60
*Strongyloides* spp.	37.24	37.54	37.24	21.92	22.52	22.22	18.02	18.62	18.62	14.71	14.41	14.11	13.51	13.21	12.31

Legend: SCP, SCC, ECP, and ECC denote southwestern city periphery, southwestern city center, eastern city periphery, and eastern city center, respectively.

**Table 2:** One-tailed *p*-values for the differences in prevalence between tested populations.

Infectious Agent	*p*-Values for the Difference Between Baboons and Human Samples	*p*-Values for the Difference Between SCC and SCP	*p*-Values for the Difference Between ECC and ECP
*Campylobacter* spp.	0.0001 *	0.0004 *	0.30
*Clostridioides* *difficile*	0.009 *	0.001 *	0.43
*Mycobacterium* spp.	0.002 *	0.01 *	0.89
*Salmonella* spp.	0.1808	0.13	0.68
*Shigella* spp.	0.004 *	0.002 *	0.61
*Staphylococcus aureus*	0.41	0.005 *	0.60
*Entamoeba histolytica*	0.009 *	0.007 *	0.56
*Giardia lamblia*	0.013 *	0.03 *	0.24
*Cryptosporidium* spp.	0.005 *	0.023 *	0.54
*Cyclospora* spp.	0.008 *	0.0044 *	0.32
*Balantidium coli*	0.0021 *	0.01 *	0.64
*Blastocystis hominis*	0.006 *	0.037 *	0.28
*Enterobius vermicularis*	0.0004 *	0.010 *	0.44
*Hymenolepis* spp.	0.0051 *	0.025 *	0.01 *
Hookworms	0.04 *	0.030 *	0.47
*Trichuris trichiura*	0.022 *	0.040 *	0.42
*Schistosoma mansoni*	0.04 *	0.90	0.84
*Strongyloides* spp.	0.038 *	0.02 *	0.18

Legend: SCP, SCC, ECP, and ECC denote the southwestern city periphery, southwestern city center, eastern city periphery, and eastern city center, respectively. An asterisk (*) indicates a statistically significant result with *p* < 0.05.

**Table 3:** *16S r*RNA gene clusters of bacteria isolated from Baha city based on NCBI BLASTN analysis.

Cluster Designation	Number	Source	*16S r*RNA Gene Similarity	NCBI Accession
*Clostridioides difficile* strain DSM 11209	1	PH (*n* = 1)	100%	X73450.1
*C. difficile* strain JCM 5256	3	PH (*n* = 1) SCP (*n* = 2)	98%	AB632386.1
*Clostridium* sp. CYP4	4	PH (*n* = 1) SCC (*n* = 1) SCP (*n* = 2)	99%	DQ479414.1
*C. difficile* strain JCM 5244	2	PH (*n* = 1) SCP (*n* = 1)	99%	AB632375.1
*C. difficile* strain M120	1	PH (*n* = 1)	100%	FN665653.1
*C. difficile* strain CD196	5	PH (*n* = 1) SCC (*n* = 2) SCP (*n* = 2)	100%	FN538970.1
*C. difficile* strain JCM 1296	1	PH (*n* = 1)	99%	AB548672.1
*C. difficile* strain VPI 10463	1	PH (*n* = 1)	99%	AF072473.1
*C. difficile* strain M68	1	SCC (*n* = 1)	98%	FN668375.1
*C. difficile* strain 630	3	SCC (*n* = 2) SCP (*n* = 1)	98%	AM180355.1
*Staphylococcus aureus* strain 502A	14	PH (*n* = 4) SCC (*n* = 6) SCP (*n* = 4)	98–99%	NZ_CP007454.1
*S. aureus* sp. NY-N1	2	PH (*n* = 2)	99%	FJ592986.1
*S. aureus* strain Y22	4	PH (*n* = 4)	99%	KF923962.1
*S. aureus* strain USA-ISMMS1	4	PH (*n* = 2) SCP (*n* = 2)	99%	NZ_CP007176.1
*S. aureus* strain Y19	2	PH (*n* = 2)	99%	KF923961.1
*S. aureus* strain P91-7354b	10	PH (*n* = 2) SCC (*n* = 4) SCP (*n* = 4)	99%	DQ647042.1
*S. aureus* strain SMKV-2	6	SCC (*n* = 4) SCP (*n* = 2)	98%	DQ306891.1
*S. aureus* strain RKA6	2	SCP (*n* = 2)	99%	EF463060.1
*S. aureus* strain GSA-51	2	SCP (*n* = 2)	99%	JN315154.1
*Campylobacter* spp. 706H	2	PH (*n* = 2)	98.7%	KF040443.1
*Ca. hypointestinalis* subsp. *hypointestinalis* stain 95-2	2	PH (*n* = 2)	98%	AB301960.1
*Ca. jejuni* subsp. *jejuni* strain 81-176	4	PH (*n* = 2) SCC (*n* = 1) SCP (*n* = 1)	98.4%	AF486558.1
*Ca. jejuni* subsp. *jejuni* strain 1182-3/95	3	PH (*n* = 2) SCP (*n* = 1)	99%	EU127533.1
*Ca. jejuni* strain 6871	2	PH (*n* = 2)	99%	AY628389.1
*Ca. fetus* subsp. *venerealis* strain 84-112	3	PH (*n* = 2) SCC (*n* = 1)	99%	HG004426.1
*Ca. lanienae* strain 24639	2	PH (*n* = 2)	99%	HM462455.1
*Ca. jejuni* subsp. *jejuni* NCTC 11168	3	PH (*n* = 2) SCP (*n* = 1)	98%	AL111168.1
*Ca. lari* strain RM2100	4	PH (*n* = 2) SCP (*n* = 2)	99%	KF855290.1
*Ca. lanienae* strain 24639	1	SCP (*n* = 1)	98.5%	HM462455.1
*Ca. coli* CVM N29710	1	SCP (*n* = 1)	98.5%	NR_121825.1
*Mycobacterium simiae*	10	PH (*n* = 8) SCC (*n* = 1) SCP (*n* = 1)	98.7–99%	X52931.1
*M. fortuitum* ATCC 49403	3	PH (*n* = 3)	99.4%	X65528.1
*M. kansasii*	4	PH (*n* = 4)	100%	M95469.1
*M. kansasii* strain DSM 44162	5	PH (*n* = 4) SCP (*n* = 1)	99.2%	NR_042164.1
*M. abscessus*	3	PH (*n* = 2) SCP (*n* = 1)	98.8%	AY360327.1
*M. simiae* sequevar Msi-A	1	PH (*n* = 1)	98%	Z46426.1
M. bovis subsp. bovis AF2122/97	5	PH (*n* = 3) SCC (*n* = 1) SCP (*n* = 1)	99%	BX248338.1
*M. gordonae* strain HA-1	4	PH (*n* = 2) SCC (*n* = 1) SCP (*n* = 1)	99.2%	KC684911.1
*M. szulgai*	5	PH (*n* = 3) SCC (*n* = 1) SCP (*n* = 1)	99.3%	M61665.1

Legend: PH, *Papio hamadryas* fecal samples; SCP, human fecal samples collected from southwestern city periphery of Baha city; SCC, human fecal samples collected from southwestern city center of Baha city.

## Data Availability

The raw data supporting the conclusions of this article will be made available by the authors on request.
